# Design and Use of a Custom Phantom for Regular Tests of Radiography Apparatus: A Feasibility Study

**DOI:** 10.3390/jimaging10100258

**Published:** 2024-10-18

**Authors:** Nikolay Dukov, Vanessa-Mery Valkova, Mariana Yordanova, Virginia Tsapaki, Kristina Bliznakova

**Affiliations:** 1 Department of Medical Equipment, Electronic and Information Technologies in Healthcare, Medical University of Varna, 9002 Varna, Bulgaria; kristina.bliznakova@mu-varna.bg; 2 Specialized Hospital of Obstetrics and Gynecology—Varna, 9002 Varna, Bulgaria; vanesavalkova99@abv.bg; 3 Training sector “X-Ray Laboratory Assistant”, Medical College, Medical University of Varna, 9002 Varna, Bulgaria; kupenova@mu-varna.bg; 4 Section of Dosimetry and Medical Radiation Physics, Division of Human Health, Department of Nuclear Sciences and Applications, International Atomic Energy Agency, 1400 Vienna, Austria; v.tsapaki@iaea.org

**Keywords:** X-Ray, quality control, radiographer, in-house phantom

## Abstract

This study investigates the feasibility of employing an in-house-developed physical phantom dedicated to the weekly quality control testing of radiographic systems, performed by radiographers. For this purpose, a 3D phantom was fabricated, featuring test objects, including a model representing a lesion. Alongside this phantom, a commercial phantom, specifically, IBA’s Primus L, was utilized. Weekly imaging of both phantoms was conducted over a span of four weeks, involving different imaging protocols and anode voltages. Subsequently, the obtained data underwent visual evaluation, as well as measurement of the intensity of selected regions of interest. The average values for three incident kilovoltages remained consistently stable over the four weeks, with the exception of the “low energy” case, which exhibited variability during the first week of measurements. Following experiments in “Week 1”, the X-Ray unit was identified as malfunctioning and underwent necessary repairs. The in-house-developed phantom demonstrated its utility in assessing the performance of the X-Ray system.

## 1. Introduction

The adoption of X-Ray imaging is experiencing rapid growth, driven by the technological advances and the enhanced diagnostic capability of evolving imaging methods [1]. While ultrasound and magnetic resonance imaging have been developed as alternative non-invasive modalities, there has still been a noticeable rise in the utilization of X-Rays [2,3,4].

X-Rays are generated by X-Ray tubes, which are an essential component of medical devices used for diagnostic purposes. In addition to the X-Ray tube, other key components of these devices include digital detectors, the patient table, and elements related to acquisition geometry. Excluding dental devices, radiography stands out as the predominant modality among these devices [5,6,7]. A crucial component of any X-Ray system is the X-Ray tube. Failures related to its rotation mechanism, anode preheating, heat dissipation, mechanical wear, electrical arcing, and inadequate insulation—especially regarding voltage and current regulation—can significantly disrupt operations and lead to costly repairs or downtime. Proper maintenance and monitoring of these aspects are essential to ensure system reliability and prevent unexpected failures [8,9,10,11]. In addition to the various potential failures in X-Ray tubes, several parameters of X-Ray images affect the quality of the visual representation or the clarity of the X-Ray images. These parameters include contrast, resolution, noise, unsharpness, size, distortion, and artefacts. All these factors are crucial for both disease screening and diagnosis [12,13,14].

Effective quality assurance (QA) has positive impacts by enhancing image quality and mitigating patient dose exposure. Quality control (QC) is an essential part of QA, and it includes the periodic and annual testing of all components of an X-Ray system. The key professional coordinating these activities in the Diagnostic Radiology department is the clinically qualified medical physicist (CQMP) [Directive 97/43/EURATOM of the EU] [15]. This includes the dosimetric control, monitoring, and assessment of the required levels of the operating parameters of the equipment, which can be defined, measured, and controlled. These are activities normally performed by a CQMP [15,16]. Unfortunately, there is a significant shortage of CQMPs with expertise in radiology compared to the sector’s growing needs [17]. Even in hypothetical situations where such professionals exist, Radiology departments lack the essential tools for them to work efficiently and stay updated on the latest developments in the field [18]. One approach to stay up to date is by following dedicated courses for Experts in Medical Physics, such as the EUTEMPE-RX course [19]. These courses provide comprehensive training in the fundamentals of both QA and QC, utilizing essential phantoms and software designed for processing X-Ray images focusing on diagnostic and interventional radiology. This provides CQMPs with the skills and competencies to attain and sustain a high level of proficiency.

Specifically, for daily and weekly QC and in order to have adequate X-Ray equipment monitoring in the everyday clinical routine, an efficient alternative is the utilization of a radiographer who is well trained to perform QC under the supervision of a CQMP [20]. With adequate training and the use of simple phantoms, the radiographer can effectively manage numerous daily or weekly tasks that fall within their competency, contributing to the successful execution of quality control measures. Locally developed low-cost and easy-to-construct phantoms, such as the IAEA phantom [21,22], as well as other low-cost, in-house-made phantoms, developed by other researchers and applicable in the field of mammography, digital radiography, computed radiography, fluoroscopy, angiography, and dental cone beam computed tomography units [18,23,24], have already been used successfully in test checks.

In this study, we tested the hypothesis that a physical phantom, designed with anthropomorphic features and made from materials closely mimicking the radiological properties of human tissue, could be used by radiographers for the weekly testing of radiography systems. A preliminary study was conducted using the abdomen X-Ray protocol, yielding encouraging results [25]. This positive outcome has prompted us to undertake a larger study involving multiple X-Ray imaging protocols, including the use of a commercial phantom to further validate the findings. The current testing was conducted by measuring the radiation dose in the phantom and evaluating the intensity of test objects placed within it, as these parameters are directly relevant to evaluating the performance of an X-Ray system.

## 2. Materials and Methods

### 2.1. Phantoms

**Figure 1:**
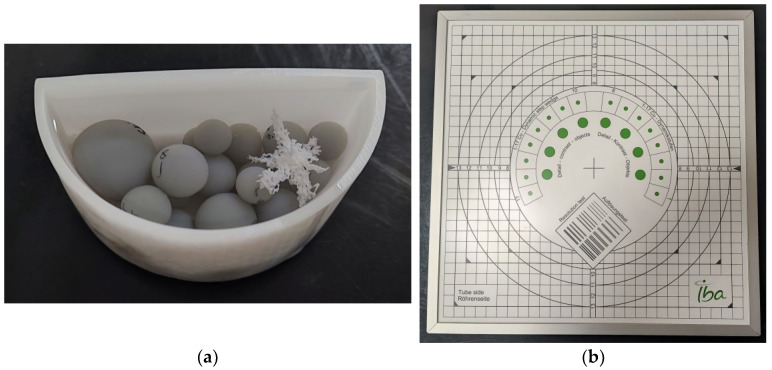
Phantoms used in the study: (**a**) in-house-developed and 3D-printed anthropomorphic phantom; (**b**) commercially available test device, Primus L by IBA.

The study included the production of a 3D physical phantom by using the 3D printing technique. Once produced, the phantom was imaged under an X-Ray radiography system, which undergoes mandatory regular QC checks. The imaging of the phantom was performed under various conditions once a week over the course of four weeks. In addition, the IBA Primus L phantom and the IBA DOSIMAX plus I certified dosimeter with a solid-state RQA detector were used in the measurements for comparison purposes. The acquired imaging data were evaluated both subjectively and objectively by processing the data to extract statistical features for assessing the radiography system. The two phantoms that were used in this study, (a) the in-house-developed 3D-printed phantom and (b) the IBA phantom, are shown in Figure 1.

The in-house-developed, 3D-printed phantom was chosen because the materials used in its construction possess X-Ray attenuation characteristics similar to those of human tissues. Additionally, it has been employed in various experiments with different setups, including both low and high anode voltages [26,27,28], demonstrating its utility in proof-of-concept studies. This physical phantom also has a computational version, allowing computer simulations to be conducted. The 3D-printed physical phantom (Figure 1a) is composed of Formlabs White Resin V4 (ρ = 1.15–1.20 g∙cm^−3^, when cured) container filled with liquid pharmacological-grade paraffin (ρ = 0.8–0.87 g∙cm^−3^), which is highly purified liquid paraffin, with consistent quality across different batches. In this phantom, 3D-printed spheres with Formlabs Grey Resin V4 (ρ = 1.15–1.20 g∙cm^−3^, when cured) and a 3D-printed tumour model printed from Formlabs White Resin V4 were added, as shown in Figure 1a. In this configuration, the container represents the skin, and the spheres serve the purpose of imitating glandular tissue. The pharmacological-grade paraffin, on the other hand, depicts fat or adipose tissue. As mentioned earlier, the phantom contains a tumour component, specifically chosen for its shape, which predisposes it to low detectability by a reader. This approach is one of the methods for the creation of anthropomorphic phantoms [29], i.e., separately printing the different phantom components with different printing methods and materials and then assembling the whole phantom.

This custom-made phantom can be classified as possessing anthropomorphic features, in line with the classification developed by the authors [30]. Specifically, its shape is anthropomorphic, resembling the compressed form of a female breast. Additionally, the manufactured lesion exhibits anthropomorphic characteristics, reflecting realistic spiculated breast lesions. Such lesions have also been studied by other researchers [31,32,33,34]. Furthermore, the materials used to print the phantom components were carefully chosen to mimic the properties of breast tissues, making the model suitable for mammography applications. All components were printed using an SLA (stereolithography) 3D printer—Formlabs Form 3—which ensured the homogeneity of the printed object. The printing materials were chosen based on their X-Ray properties, which are close to the properties of glandular and adipose tissue, and they were measured to determine various energies [35,36]. The resolutions of the printed layer for the container, the lesion, and the spheres were set to 100 µm, 50 µm, and 50 µm, respectively.

**Figure 2:**
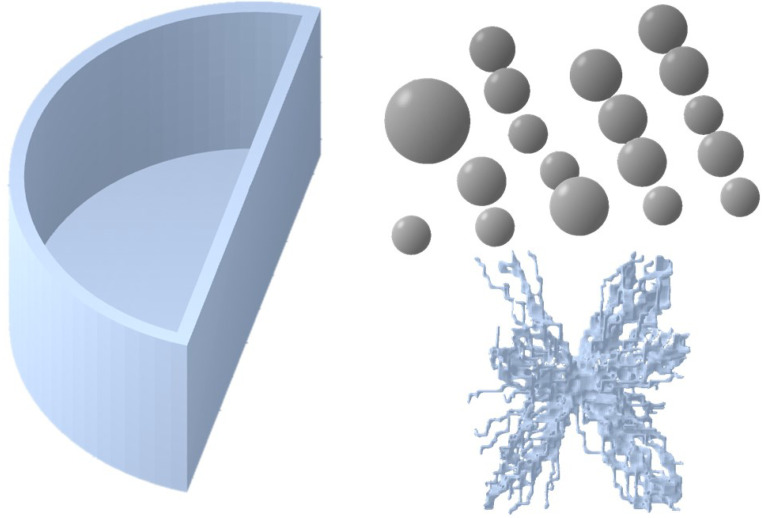
Three-dimensional models used as templates for printing the components of the in-house anthropomorphic phantom. The semi-cylinder container and spheres were designed using DesignSpark Mechanical, while the lesion model was generated based on a mathematical algorithm and sourced from the Maxima database [31].

Specifically, the bases of the physical phantom parts were their computational models, as shown in Figure 2. The semi-cylindrical container with a size of 10.0 cm × 5.0 cm × 4.5 cm and the 18 spheres with radiuses, listed in Table 1, were modelled using a free version of DesignSpark Mechanical v6.0.

The tumour model was taken from the Maxima database (https://zenodo.org/records/7099532, accessed on 10 March 2023), which contains breast lesion models with realistic shapes, the source of which is from both patient image segmentations and computer-based mathematical models [31,37]. A mathematically generated model with an irregular shape was chosen for this study. The model generation included two basic steps: initial shape formation by random walks, followed by solid tumour formation.

### 2.2. Imaging Protocols

**Figure 3:**
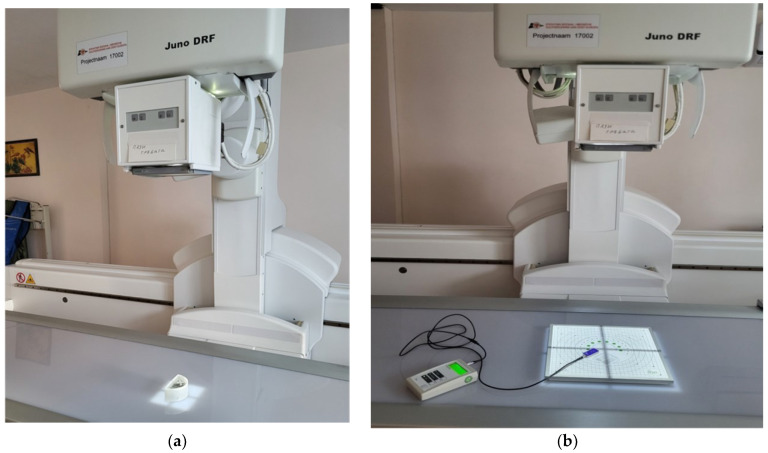
Imaging scenarios performed with the phantoms—(**a**) the 3D-printed, in-house-developed phantom and (**b**) Primus L—using the Philips Juno DRF.

The developed 3D-printed phantom was used in weekly imaging evaluations with a Philips Juno DRF (Figure 3) by using the digital radiography mode. In a period of four weeks, the 3D-printed phantom and IBA’s Primus L phantom were imaged once per week under identical imaging conditions by using the Abdomen Debout F protocol. Further, to test the capabilities of the in-house phantom, the latter was evaluated for the same testing period under three imaging protocols, as summarized in Table 2. With a given protocol, a single image was taken at three different anode voltages in AEC mode. The detector of the X-Ray equipment was PIXIUM RF4343 with a pixel matrix of 2880 by 2881 and a 148 μm pixel pitch. The focus-to-detector distance in all cases was kept at 110 cm.

### 2.3. Data Processing and Evaluation

**Figure 4:**
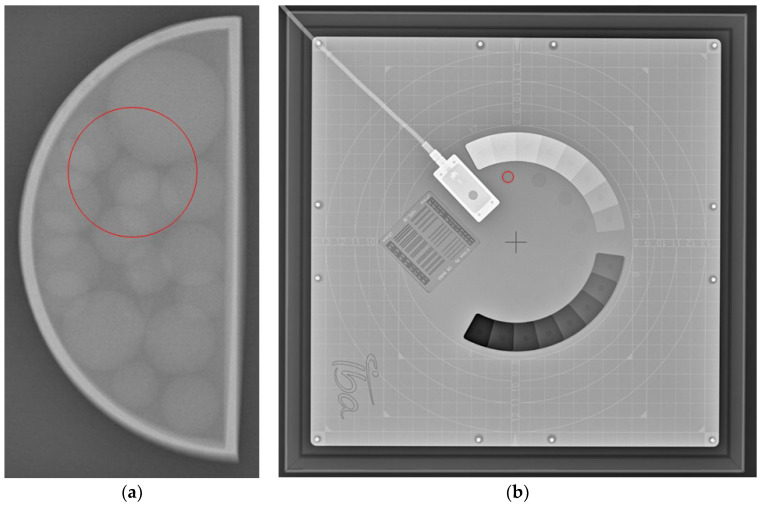
Example of the area of ROI (circle in red) taken for (**a**) the 3D-printed phantom and for (**b**) IBA’s Primus L phantom.

The objective (qualitative) analysis included the measurement of the intensity of the images by using ImageJ v1.54f [38]. The tool used for creating the region of interest (ROI) was the “Oval” tool, with known dimensions and areas, which were applied to all of the studied images. The size and position of the ROI were carefully chosen to avoid covering the tumour area. The mean intensity value of the pixels, as well as the standard deviation, was then calculated. Similarly, the mean intensity values with standard deviation for IBA’s Primus L phantom were also calculated. The ROI used for measuring the intensity values is demonstrated on the radiography images in Figure 4. The resulting values calculated from the ROIs of both phantoms from the four weeks and the three energies used with Abdomen Debout F were plotted and compared.

The subjective (qualitative) evaluation consisted of the visual assessment of the visibility of objects in the phantoms. For the in-house-developed phantom, this translated to the clear distinguishment of the various spheres and the single tumour entity through the evaluation period. In the case of the Primus L phantom, it included counting the number of visible low-contrast objects from the phantom every week. Furthermore, the phantom included a bar pattern for measuring the limiting spatial resolution of the system in terms of lines per mm (lp/mm), which was also evaluated visually during every one of the test weeks.

## 3. Results and Discussion

**Figure 5:**
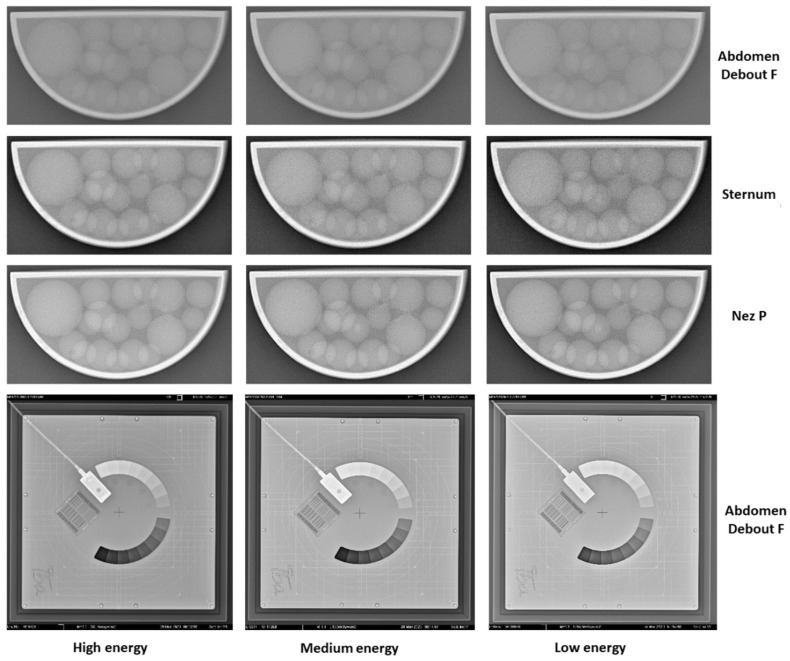
X-Ray images from week 3 of the performed 4-week study with the 3D-printed phantom filled with liquid paraffin. The shown images are from the three used protocols (Abdomen Debout F, Sternum, and Nez P) and the three respective energies used (high, medium, and low energy in accordance with Table 2). Furthermore, X-Ray images of the same period for the Primus L phantom are shown as well.

Radiography images of the 3D-printed phantom for the three imaging protocols are shown in Figure 5. In the same figure, the radiography images of the IBA Primus L phantom are displayed. The effects of the different energies on the visibility of the objects can be observed. Phantom images were visually compared over a four-week period, and no noticeable differences were observed between the images from the first week and those from the following weeks.

**Figure 6:**
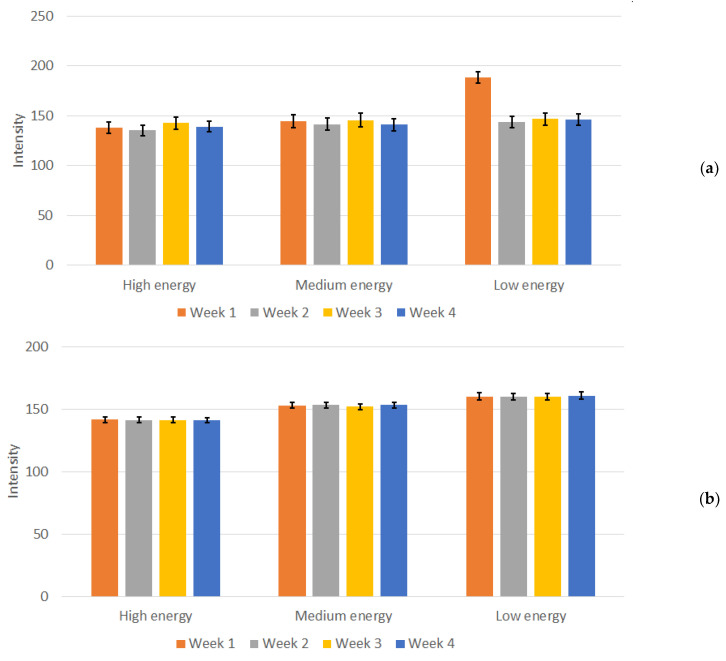
Measured mean intensity values and calculated standard deviation for (**a**) the in-house-developed phantom and (**b**) the Primus L phantom for the Abdomen Debout F protocol.

Figure 6 summarizes the resulting image intensity measurements for the Abdomen Debout F protocol for both phantoms for the period of evaluation. In the measurements conducted for the Abdomen Debout F protocol (Figure 6a), it was observed that the standard deviation was the lowest in terms of values, compared to the rest of the measurements of the other considered protocols. An analysis of the Abdomen Debout F protocol measurements of the intensity revealed that the standard deviation for high, medium, and low energy levels was within the ranges of ±5 to ±6, ±6 to ±7, and ±6, respectively. Furthermore, a coefficient of variation (CV) was calculated, resulting in values of <5% for the Abdomen Debout F protocol, commonly deemed as acceptable [39].

**Figure 7:**
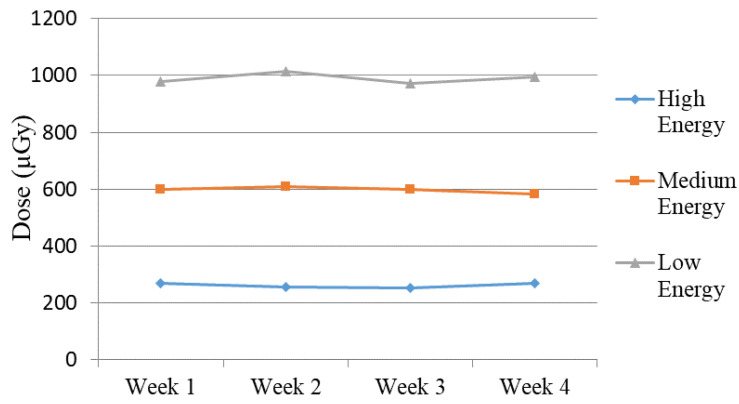
Measured dose for Abdomen Debout F protocol (high, medium, and low energy) during the four weeks of the study.

Moreover, the average value obtained over the four weeks for the three energies remained consistent. Specifically, the values exhibited consistency across the four weeks of testing, with values fluctuating between 135 and 143 for high energy, 141 to 146 for medium energy, and 143 to 147 for low energy. An exception for “low energy” occurred during the first week of measurements, with a mean value of 188. This deviation is evident in Figure 6a. During the experiments performed in “Week 1”, the X-Ray unit was found to be malfunctioning, specifically the AEC system, when low energy settings were used, as shown from the experimental data, and the unit was subjected to repairs. After the repairs, no noticeable deviations in the intensity of the images (both from numerical measurements and from visual inspection) and the performance of the unit were reported in the following weeks. In contrast, this distinction was not observed when employing other protocols and energy settings with this 3D-printed phantom as well as with the Primus L phantom (Figure 6b). This points to a settings-dependent issue and highlights the importance of performing tests under various conditions. The weekly measured dose, depicted in Figure 7, illustrates the normal functioning of the X-Ray apparatus as well.

**Figure 8:**
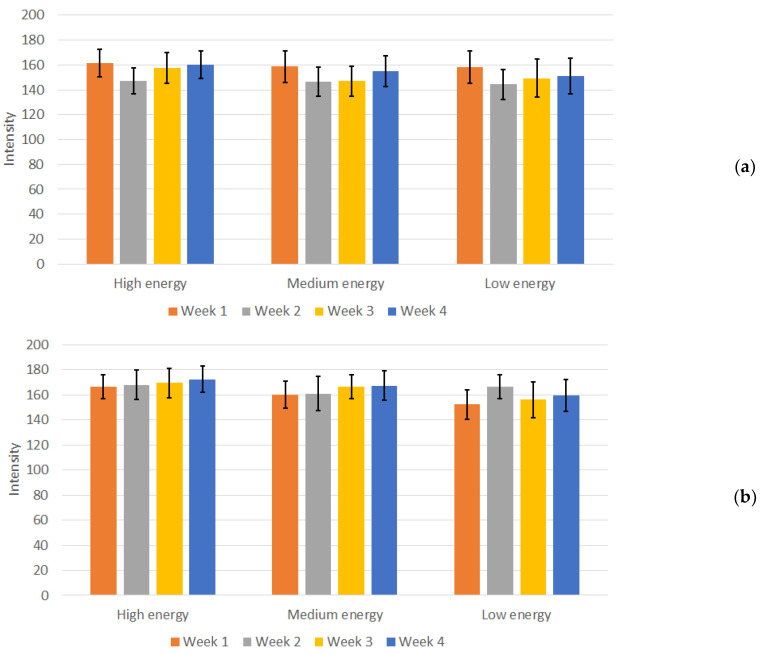
Mean intensity and standard deviation for (**a**) Sternum protocol (high, medium, and low energy and (**b**) Nez P protocol (high, medium, and low energy) measurements during the four weeks of the study.

The resulting measurements of the image intensities for the 3D-printed phantom with the remaining two protocols, across the three energy levels, over the duration of four weeks, are presented in Figure 8.

In contrast to the measurements in the Abdomen Debout F protocol, in both the Sternum and Nez P protocols, the calculated deviations from the mean intensity values for the 3D-printed phantom were prominent, as there were variations in mean intensity over the weeks. The CV was calculated for these protocols as well, resulting in values between 6% and 10%, depending on the protocol and energy used. Although slightly higher, this CV can also be considered acceptable, given the heterogeneous region from where the measurements were conducted and the increased sharpness of the images, as seen from Figure 5. Furthermore, no visual difference and changes in the visibility of individual structures in the images of the measured phantom were observed.

**Figure 9:**
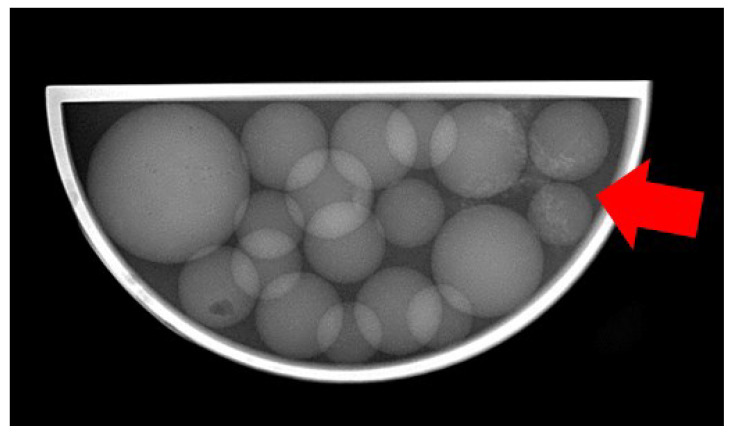
Radiography image of the 3D-printed phantom made with the Nez P imaging protocol at 75 keV, with visible structures of the tumour entity (red arrow).

As a consequential outcome of the conducted measurements, optimal imaging conditions were identified for the highly challenging low-contrast tumour entity within the utilized phantom. Specifically, the Nez P imaging protocol proved effective with settings of 75 kVp anode voltage, 10 mAs, and a 25 ms exposure time, which resulted in the radiography image shown in Figure 9.

The time needed to print the container and the spheres was approximately 3 and 5 h, respectively. For the container, we needed 45 mL of resin, while for the spheres, a total of 53.13 mL of resin was needed. The physical tumour entity required 7.63 mL of resin used and 4 h and 4 min of printing. Overall, the cost of the prepared phantom is around EUR 20 in terms of materials, including the liquid paraffin.

The primary constraint in this study arises from the utilization of an anthropomorphic physical phantom used to test various breast imaging setups with a general-purpose radiography machine. This choice was driven by several practical considerations. Firstly, commercially available anthropomorphic phantoms designed for image quality assessments, including whole-body, abdominal, chest, and head phantoms, come with a substantial price tag ranging from USD 4000 to USD 10,000, contingent on the imaging modality and simulated anatomy [40]. To address this, the research team is actively exploring the creation of a cost-effective pediatric phantom using 3D printers [41]. This pediatric phantom will be instrumental in further testing of the imaging procedures. Secondly, the chosen anthropomorphic phantom, with its inclusion of irregular lesion models and low-contrast objects, presented an opportunity to evaluate its performance in comparison to commercially available IBA test phantoms. Nevertheless, the use of anthropomorphic breast phantoms with general-purpose machines is not unique to this study. Other research endeavours, such as those implementing proof of concepts like dual energy, have similarly reported leveraging this approach [42,43,44,45,46].

In this study, our primary objective was to measure the radiation dose and the intensity of details using the custom-made phantom, as these parameters are directly relevant to assessing the performance of the X-Ray system and are easily assessed by the radiographer. Comprehensive quality assurance includes the evaluation of additional parameters such as noise, contrast scale, spatial resolution, sensitivity, spatial uniformity, and linearity. This requires specialized spatial phantoms and is performed by a certified company that regularly conducts quality control on the radiography system. These phantoms incorporate test objects designed specifically to evaluate each parameter, such as contrast scales and spatial resolution grids. However, the fabrication of such complex test objects in-house presents significant challenges.

The preliminary results obtained create possibilities for conducting tests on both the new system currently under development for dedicated breast CT at our university and the existing general-purpose system. Data collection is planned over the next 12 months, providing valuable insights. In addition, another limitation is the lack of a digital mammography system in our university hospital. We are currently in the process of acquiring a new digital tomosynthesis system, where the new phantom is planned to be used together with a commercial phantom.

## 4. Conclusions

This study examined the efficacy of a specially designed in-house phantom on a planar radiography system. Over the course of four weeks, the phantom was effectively employed to assess the performance of the X-Ray unit, revealing promising results in terms of quality control. To deepen our understanding, further investigations will be conducted, incorporating new scenarios for testing, as well involvement in the initiative of IAEA for remote quality control for radiography will be realized. These efforts aim to explore the broader suitability and applicability of the phantom within the workflow of radiographers.

## Figures and Tables

**Table 1:** Description of the printed physical spheres.

Radius, mm	13.0	9.0	8.0	7.5	7.0	6.0
Number of spheres	1	1	1	5	3	7

**Table 2:** Used clinical protocols and kV settings in the study.

Imaging Protocol			General Characteristics of the Selected Imaging Protocols
Abdomen Debout F			The protocol is used for abdominal imaging and for hysterosalpingography. The latter is applied for diagnosing the complete or partial blockage of the fallopian tubes and the detection of various pathological conditions affecting the uterus. It is also used for diagnosing various diseases of the kidneys, bladder, and ureters.
High energy	70 kV	200 mA	
Medium energy	66 kV	200 mA	
Low energy	55 kV	50 mA	
Sternum			The protocol is used for imaging the sternum, particularly when there is a suspicion of a sternum fracture. Given the challenge of visualizing the breastbone (sternum) adequately in a frontal view, a lateral profile is often employed for a more comprehensive examination.
High energy	80 kV	200 mA	
Medium energy	70 kV	125 mA	
Low energy	60 kV	125 mA	
Nez P			The protocol is used for nasal imaging and involves examining the nose and sinuses for various purposes, including the detection of fractures, post-rhinoplasty preventive imaging, and the identification of sinus issues such as sinusitis and facial bone polyps.
High energy	46 kV	6.3 mA	
Medium energy	44 kV	4 mA	
Low energy	40 kV	3.2 mA	

## Data Availability

Data that supports this study are available at http://doi.org/10.5281/zenodo.13260069, accessed on 8 August 2024.
